# High‐pressure freezing of mechanically stretched cells

**DOI:** 10.1111/jmi.13411

**Published:** 2025-04-02

**Authors:** Edward Felder, Jan L. Rüth, Bassam Abu‐Omar, Martin Wohlwend, Paul Walther, Clarissa Read

**Affiliations:** ^1^ Institute for General Physiology Ulm University Ulm Germany; ^2^ Engineering Office M. Wohlwend GmbH Sennwald Switzerland; ^3^ Central Facility for Electron Microscopy Ulm University Ulm Germany

**Keywords:** cell stretch, elastic growth substrate, high pressure freezing

## Abstract

High‐pressure freezing (HPF) is an electron microscopy (EM) preparation technique with superb ultrastructural preservation. Combined with EM tomography it provides virtual EM serial sections with extraordinary spatial resolution. For HPF, cells are usually cultured on a rigid sapphire disc that provides a tight fit in the holding bracket of the HPF apparatus. Since we are using extensible elastic silicone membranes as a growth support to perform cell stretch experiments, we developed a method to clamp the stretched silicone membrane and place it instead of the sapphire disc into the HPF holding bracket. Compared to chemical fixation the HPF immobilised cells showed improved structural preservation, partly even on a molecular level. However, the outstanding quality of HPF immobilised cells on sapphire discs was not achieved. Moreover, regions with obvious freezing artefacts seemed to be more abundant in the HPF silicone membranes, probably caused by lower heat transfer rates of the silicone membrane during the HPF process.

Taken together, we have shown that HPF immobilisation can be performed on growth supports different than sapphire discs. Since even stretched membranes can be used with the new method, also other unconventional growth supports should not pose a problem.

## 1 INTRODUCTION

Most cells are subjected to mechanical forces to a certain degree. Besides developing reinforcement structures that maintain cellular structural integrity, evolution also developed mechanisms to detect mechanical stimuli, interpret them as environmental clues and respond accordingly. Such mechanisms include fast activation of ion channels (e.g. deflecting stereocilia in the vertebrate inner ear; for review, see Qui et al.^1^) as well as slow processes via mechanically activated signalling cascades with response times of days or more (e.g. osteoblasts and bone remodelling, for review, see Hadjidaki and Androulakis^2^ and Rosa et al.^3^). The scientific focus of our group rests on keratin filaments (KF), the epithelial subtype of intermediate filaments, and on desmosomes, that anchor KF at the cell border and connect KF of adjacent cells. Classically this transcellular KF‐network is described as a passive (although dynamic) load bearing structure.^4^ However, mechanical stimulation can induce KF‐phosphorylation^4,12^ and we found stretch‐induced phosphorylation of KF on multiple serin and threonine residues within fractions of a minute after cell stretch of keratinocytes.^5^ Since KF phosphorylation alters the mechanical properties of the cell, we interpret this stretch‐induced KF‐phosphorylation as a mechano‐protective mechanism, possibly to avoid harmful tensile stress on the desmosomes.

The molecular sensor that initiates a signalling cascade leading to the above mentioned KF phosphorylation has not been identified yet. However, a mechanosensor within the desmosomes seems ideal for this purpose with desmoplakin being the most promising candidate. Desmoplakin provides the physical link between KF and the other desmosomal components, all of them forming the so‐called dense plaque, an electron dense disc characteristic for desmosomal ultrastructure. In line with this hypothesis, it has been shown that (computational) extension of desmoplakin eventually leads to unfolding of the spektrin repeat domain 5 of the protein, thereby revealing an SH3 domain.^6^ SH3 domains are known to serve as scaffolding platforms allowing other proteins to dock and hence might be a promising candidate to initiate a signalling cascade.

Considering the massive molecular dimension of desmoplakin, its distinct location at the inner surface of the dense plaque and the extraordinary extent of its (calculated) stretch‐induced extension, we expect differences in the desmosomal ultrastructure when comparing stretched and unstretched cells. This scientific question was the incentive for developing a new method to visualise desmoplakin at best possible resolution. However, this study was not intended to reveal such differences but to evaluate the new method and utilise it in upcoming experiments with sufficient sample sizes.

From an experimental point of view, it is considerably more complicated to mechanically stimulate a cell compared to more conventional, for example, pharmacological, stimulation. Thus, for studies on stretched cells, elaborate experimental setups are required. Usually, cells are cultivated on transparent and highly elastic silicon membranes (Polydimethylsiloxane, PDMS) and by extension of the membrane surface area also the attached cells are stretched. In the above‐mentioned studies we clamped the PDMS membrane between two holders (see also Section 2) and applied unidirectional stretch on the entire membrane, either with a manually operated or custom designed computer‐controlled stretching device.^5^ The ultrastructure of desmosomes with the above mentioned desmoplakin had been investigated with this technique using conventional chemical fixation.

However, optimal ultrastructural preservation of the cells would be required to observe stretch‐induced changes in the desmosome. Thus, high‐pressure freezing (HPF) followed by freeze substitution seemed the method of choice since it provides optimised ultrastructural preservation compared to aldehyde fixation and dehydration and heavy metal staining at room temperature. Besides superior structural preservation, the combination with EM tomography would also allow analysis of an almost 10‐fold larger sample thickness and tomographic 3D‐reconstruction with almost isotropic resolution of a few nm and thus, the extraction of consecutive virtual sections of a few nanometre thickness from the tomogram. Regular HPF protocols require cultivation of cells on a small (few millimetres) and rigid sapphire discs. For the experiments with stretched cells, we developed a custom‐made clamping fixture that not only allows to hold and transfer a piece of stretched membrane to the small HPF holder but also to maintain the tension of the stretched membrane throughout the entire HPF and freeze substitution and embedding procedure. The present study introduces and describes this new experimental approach and evaluates the quality of ultrastructural preservation by comparing stretched high‐pressure frozen cells with high‐pressure frozen cells grown on the sapphire disc and with stretched cells after conventional chemical fixation.

## 2 MATERIAL AND METHODS

### 2.1 Cell culture

SCC‐25 cells (derived from esophageal cancer, ACC 617, DSMZ, Leibzig, Germany) were cultivated under conventional cell culture conditions at 37°C and 5% CO_2_ in 50 mL cell culture flasks and were fed 2–3 times per week with DMEM/Ham`s F‐12 (Biochrom GmbH/Merck, Berlin, Germany) supplemented with 10% heat inactivated fetal bovine serum, 500 U/mL Penicillin + 500 µg/mL Streptomycin, and 20 mM L‐Glutamine (all: Gibco/ Fisher Scientific GmbH, Schwerte, Germany). Cells were split when confluency was reached. Seeding on PDMS membranes (SMI specialty manufacturing inc., Saginaw, USA) or the sapphire discs (3‐mm diameter, 160 µm thickness; Wohlwend GmbH) was performed 2–3 days after the last splitting at a density of ca. 80,000 cells/cm^2^ for the membranes. To enable cell adhesion the membranes had previously been coated with fibronectin (10 µg/mL in PBS for 4 h at 4°C; Sigma‐Aldrich Chemie GmbH, Taufkirchen, Germany).

Then cells were cultivated on the different growth supports for another 24–36 h until cell stretch and/or EM sample preparation was performed.

### 2.2 Cell stretch procedure

**FIGURE 1:**
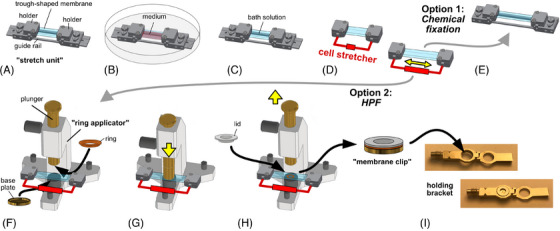
Cell stretch and fixation with PDMS membranes. The PDMS membrane was clamped by two bipartite holders to form a trough (A), which together with the guide rail is referred to as ‘stretch unit’. After autoclaving the stretch unit, the trough was filled with medium and the cells were seeded (B). For the cell stretch the medium was exchanged with bath solution (C) and the stretch unit was mounted on a manually operated cell stretcher (delineated as a red bracket). After removal of the guide rail the cell stretch was performed (D) and the cells were either (option 1) chemically fixed (E) by immediately exchanging the bath solution with fixative and then returning the membrane to the readjusted guide rail or (option 2) further processed for HPF (F). For HPF the membrane was positioned under the plunger which had been equipped with the ring (see also Figure 2 for details). The base plate was placed underneath the membrane. By lowering the plunger (G), the stretched membrane was clamped between base plate and ring and the plunger was raised again. After adding a drop of hexadecane, the lid was placed on the ring and the entire tripartite ‘membrane clip’ (H) was excised and put in the holder of the HPF device (I).

For stretching the epithelial cells, a manual stretching device was combined with the custom‐made membrane clamping device (previously described in Gerstmair et al.^7^). Briefly, a 10 mm × 80 mm rectangular piece of PDMS membrane was excised from the manufacturers PDMS sheets and clamped in the two bipartite holders. Each of the two holders consists of two accurately fitting aluminium blocks that shape the membrane to a flat‐bottomed trough between the two holders. The blocks are held in place by a guide rail. The entire assembly is referred to as the ‘stretch unit’ (see Figure 1A). To avoid leakage, a thin layer of conventional silicon glue (Otto seal S69, Herman Otto GmbH, Fridolfing, Germany) was applied along the edges where the holders touch the membrane. After the glue had dried, the stretch unit was wrapped in aluminium, autoclaved and eventually put in a 15 cm Petri dish (Sarstaedt, Nuembrecht, Germany) for cell culture (Figure 1B). Then the PDMS membrane trough was coated with fibronectin and cells were seeded (see above).

Before starting the cell stretch procedure, the medium was exchanged with bath solution (140 mM NaCl, 5 mM KCl, 2 mM CaCl2, 1 mM MgCl2, 5 mM Glucose, 10 mM HEPES pH 7.4, all: Sigma‐Aldrich Chemie GmbH, Taufkirchen, Germany; Figure 1C) to avoid alkalisation of the medium under atmospheric condition. Then the stretch unit was mounted on the stretcher (symbolised as a red line in Figure 1D) and the guide rail was removed. To avoid premature mechanical stimulation of the cells, the stretch unit and holder are designed to perform this step without any mechanical stress on the membrane, for example, by torsion of the holders. For cell stretch the distance between the two holders was manually increased at a rate of approximately 10% stretch per second to yield stretch amplitudes of 20%, 40% and 80%.

### 2.3 Preparation of stretched membranes and sapphire disc for electron microscopy

For conventional chemical fixation of the membranes the bath solution was carefully exchanged with fixative immediately after stretch with the membrane still mounted to the stretcher (Figure 1E). After fixation for 1 h in 2.5% glutaraldehyde (Plano, Wetzlar, Germany) and 1% sucrose (Sigma/ Merck, Darmstadt, Germany) in PBS the membrane was put back into the guide rail (Figure 1E), that had been adjusted to the increased length matching the stretch amplitude. Then postfixation was performed with 2% OsO_4_ (Chempur, Karlsruhe, Germany) for 1 h. After dehydration the cells were enblock stained with saturated uranyl acetate in ethanol for 30 min at 37°C, and the entire trough was filled with epoxy resin EMbed‐812 (Science Services, Munich, Germany) and polymerised for at least 48 h at 60°C. The trough‐shaped block of resin was removed from the stretch unit, cut in pieces with a hand saw and ultrathin sections (ca. 70 nm) were cut on an EM UC7 ultramicrotome (Leica microsystems, Wetzlar, Germany) and observed in a JEM1400 transmission electron microscope (JEOL. Tokio, Japan) at 120 kV acceleration voltage equipped with a Veleta camera (EMSIS, Münster, Germany).

To perform the HPF immobilisation, we needed to engineer a clamping device that allowed us to mount the membrane into the bore of the HPF‐holder with a diameter of 6 mm and a height of 1.3 mm.

**FIGURE 2:**
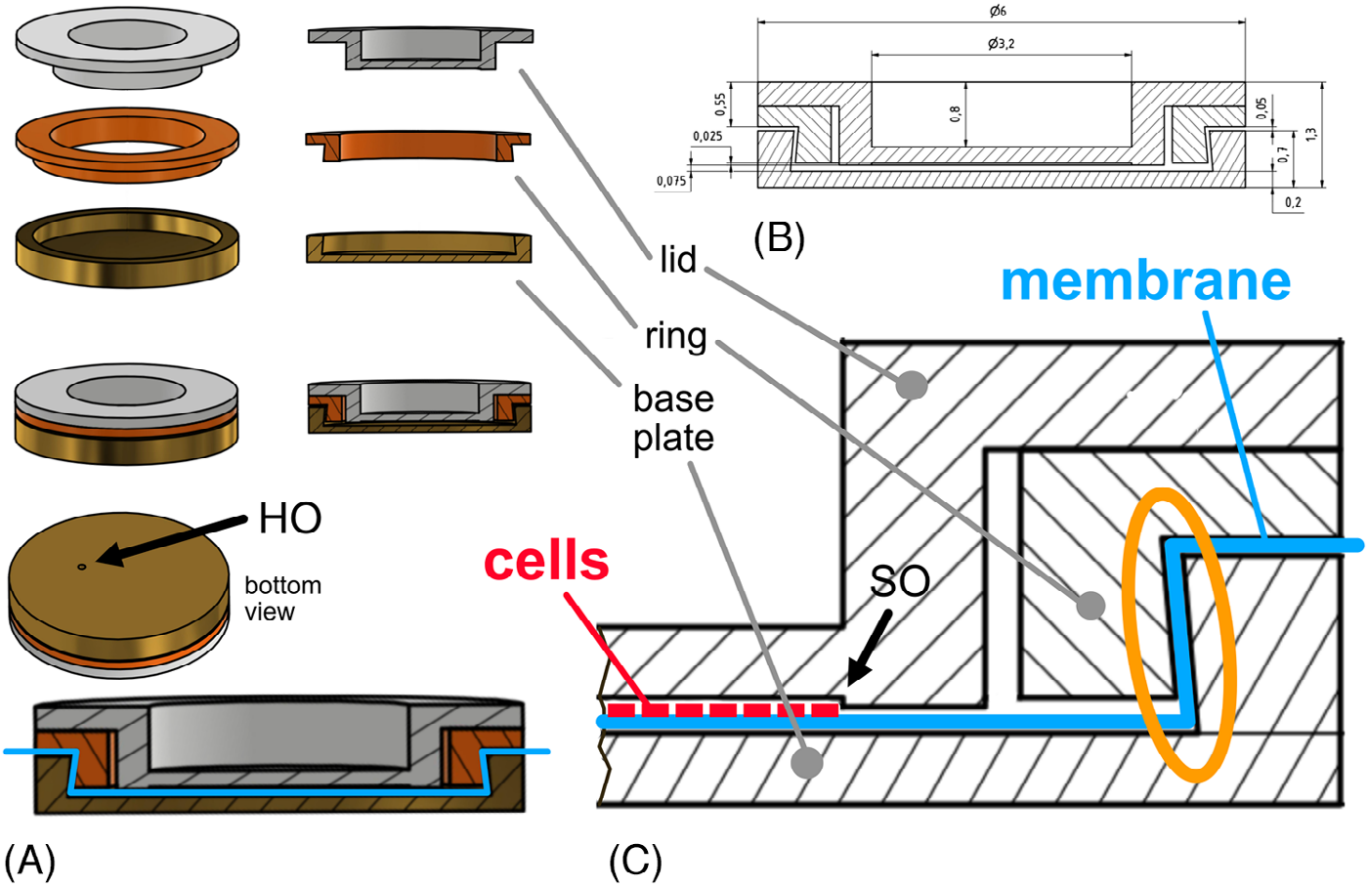
Design of the membrane clip. (A) The clip consists of three circular aluminium parts: The *base plate*, the *ring* that clamps the membrane and the *lid* that covers and protects the cell layer when HPF is performed (see also text and Figure 1 for procedural details). Dimensions and details are shown in B. The backlog of ring/base plate (see orange oval in C) fixates the membrane so that even stretch amplitudes of 80% are maintained. Note the 25 µm set off (SO) in the lid that provides enough space for the cell layer while keeping the distance to the lid small enough for fast thermal transfer during HPF. To avoid bulging of the membrane during clamping, a hole (HO) in the base plate (diameter ca. 0.5 mm) allows trapped air to escape from the space between membrane and base plate.

Immediately after cell stretch the membrane in the stretch unit with the cell stretcher still attached was transferred to a device referred to as the ‘ring applicator’ (see Figure 1F). The stretch unit was positioned in this ring applicator in a way that the sample's region of interest was centred between the plunger and the aluminium base plate. An aluminium ring had been attached to the plunger. Then the ring and the base plate were carefully pressed together by lowering the plunger so that the stretched membrane was firmly clamped between them (see Figure 1G). When lowering the membrane onto the base plate and pressing the ring down in this process, an air pocket would form between membrane and base plate. Hence, the base plate has a 0.5 mm hole (HO in Figure 2A) to release trapped air. For more details and dimensions of the components, refer to Figure 2 and Section 2.

After lifting the plunger (Figure 1H) a drop of hexadecane (Merck KGaA, Darmstadt, Germany) was added on top of the clamping ring and an aluminium lid was put on (Figure 1H). The lid provides protection of the cell monolayer against the burst of the high‐pressure liquid nitrogen. It was also designed to provide additional pressure during the immobilisation process by touching the membrane in its outer circumference, whereas a 25 µm set off in the inner circular zone (SO in Figure 2C) provides enough space for the cells. After attaching the lid, the entire assembly (referred to as ‘membrane clip’) was carefully excised from the surrounding membrane with a scalpel. Previous tests with pen markings on the membrane had shown that the stretch of the membrane is safely maintained by this approach. Then the membrane clip was transferred to the holding bracket of the HPF device (custom made by Wohlwend GmbH to fit the membrane clip; Figure 1I) and cryo‐immobilised in a Wohlwend HPF compact 01 high‐pressure freezer (Engineering Office M. Wohlwend GmbH). Prior to freeze substitution, the lid of the membrane clip (still submersed in liquid nitrogen) was removed to optimise access of the substitution solution.

For HPF of the sapphire discs, the discs with the cells were mounted in the conventional holding bracket as provided by the manufacturer as described^8^ and also cryo‐immobilised in the Wohlwend HPF compact 01 high‐pressure freezer device as described in Bergner et al.^9^

Both, the membrane clips with the stretched PDMS membranes (but with the lid removed) and the sapphire discs were freeze‐substituted in 0.2% (v/v) osmium tetroxide and 0.1% uranyl acetate (w/v) in acetone with 5% (v/v) water.^10^ The original protocol had been modified by reducing the osmium concentration to 0.2%.^11^ For that, the temperature was raised exponentially from −90°C to 0°C during 17 h, held at 0°C for 1 h and eventually raised to room temperature within 1 h. Samples were washed thrice with acetone, stepwise embedded in increasing concentrations of EMbed‐812 epoxy resin (30%, 60% and 100%, Science Services, Munich, Germany) in acetone and polymerised at 60°C for at least 48 h. After removing the sapphire discs or the base plate of the membrane clips, respectively, the cells remained in the resin block for sectioning.^11^

The most delicate step in the clamping process described above is the insertion of the ring into the baseplate (see also Figure 1G) (see also Section 4). A crucial structural detail in this regard is the backlog (orange oval in Figure 2C) of the base plate and ring, respectively. The backlog of both components needs to be tight enough to hold the clamped membrane—even when stretched. On the other hand, the backlog must provide enough space to insert the ring into the base plate without shearing off the membrane (see also Section 4).

### 2.4 Electron tomography

For obtaining virtual, only a few nm thick serial sections of the desmosomes, scanning transmission electron microscopy (STEM) tomography was performed. For that, samples were prepared as described previously.^11^ In brief, 700 nm thick sections were cut from the resin block. Sections were then collected on copper grids with parallel bars, immersed in a colloidal gold suspension for application of fiducial markers and finally coated with a 5 nm carbon layer (BAF 300, Balzers, Lichtenstein). STEM data were acquired with a JEM‐2100F electron microscope (Jeol) operating at 200 kV acceleration voltage. A tilt series of 97 STEM bright‐field images was recorded from −72° to 72° at 1.5° increments. Tomograms were reconstructed using the IMOD software package.^12^ Image processing was performed in ImageJ and affinity designer.

## 3 RESULTS

**FIGURE 3:**
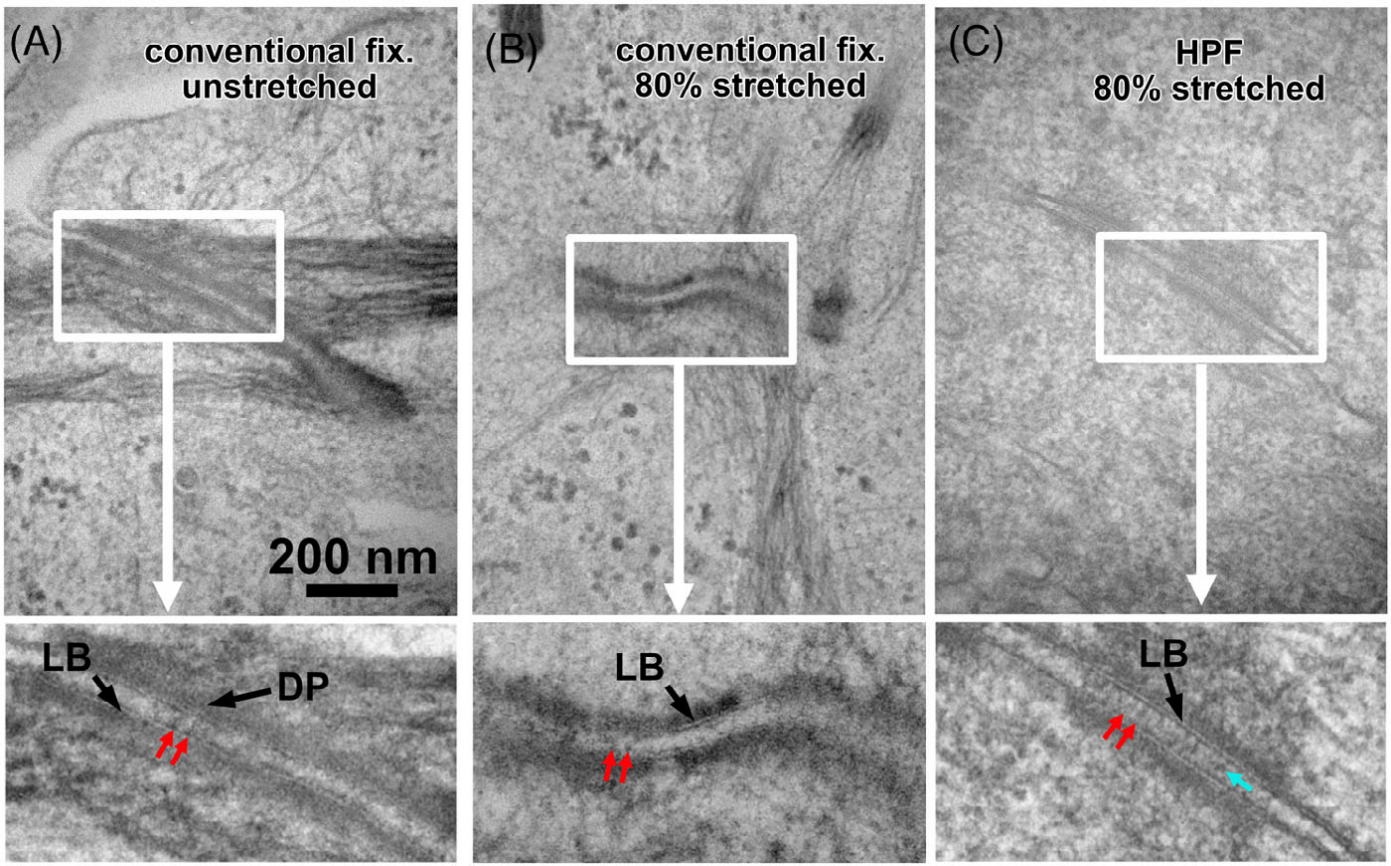
Comparison of structural preservation in conventional ultrathin sections. Desmosomes from chemically fixed cells, cultured on an unstretched membrane (A) and a membrane stretched by 80% (B). (C) A desmosome of HPF cells after 80% stretch. White rectangles indicate the regions that are shown below the panels at higher magnification. Chemical fixation (A and B) shows homogenous decent structural preservation throughout the entire sample. The two electron dense lines of the lipid bilayer (LB) and occasionally cadherins in the intercellular space (red arrows) can be distinguished. The dense plaque is indicated with DP. In the HPF specimen (C), the lipid bilayer (LB) as well as cadherins the intercellular space are more distinct and even the overlapping parts can be recognised (cyan arrow). Scale bar in A applies for all upper panels in A, B and C.

**FIGURE 4:**
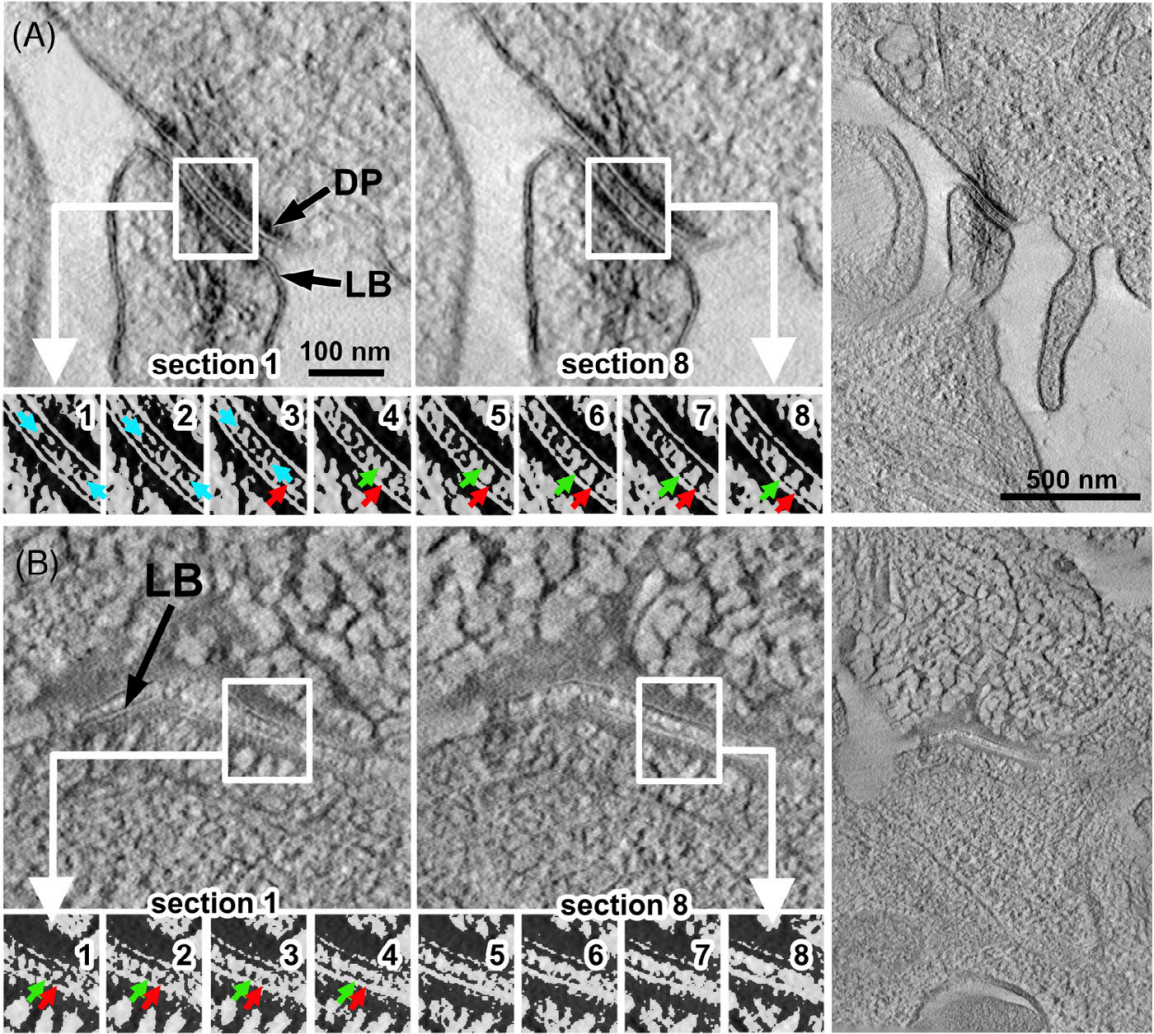
Comparison of structural preservation in virtual tomographic sections. (A) Two different virtual sections (thickness: 3.8 nm) from a tomographic reconstruction of HPF cells on a sapphire disc and (B) from cells on an unstretched PDMS membrane are shown. Overviews are depicted on the right panels and correspond to section 8 (see below). Underneath the panels with higher magnification grey‐value threshold images focus on structural details. These images stem from 8 consecutive virtual sections including the two images in the panels above. Their position is indicated by white rectangles. HPF on the sapphire disk (A) shows excellent ultrastructural preservation and no freezing artefacts. The two lines of the lipid bilayer (LB) adjacent to the dense plaque (DP) can clearly be distinguished. Also, cadherins bridging the intercellular space (red and green arrows in the threshold images indicate the same cadherin structure in different virtual sections) and the overlapping parts of opposing cadherins in the intercellular space are well preserved (cyan arrows in the threshold images). HPF of membrane (B) often led to the honeycomb‐shaped mesh structure, revealing severe freezing artefacts in the upper cell whereas fixation in the lower cell is better. Also here, cadherins in the intercellular gap can be distinguished and tracked over several sections (red and green arrows); however, the superb image quality of HPF on sapphire disc (B) was not achieved. Scale bar in A (left top panel) applies for the upper panels in A and B, scale bar in A (right panel) applies for both overview images.

Three different sample preparation procedures have been used in our study: (i) conventional chemical fixation of (stretched) cells grown on PDMS membranes, (ii) HPF and freeze substitution of cells grown on sapphire discs, a commonly used growth support for that purpose, and (iii) HPF of cells grown on a stretched PDMS membrane. Micrographs from regular ultrathin sections are compared in Figure 3 whereas virtual sections from EM‐tomograms are compared in Figure 4.

### 3.1 Comparison of regular ultrathin sections

Figure 3 shows desmosomes of unstretched (Figure 3A) and 80% stretched cells (Figure 3B), both after conventional chemical fixation (see Section 2) and a desmosome after 80% stretch after preparation by HPF (Figure 3C). In contrast to the virtual section from EM tomography in Figure 4 (thickness: 3.8 nm), the much bigger physical section thickness (ca. 70 nm) often contains even obliquely inserting keratin filaments (KF) that can be traced throughout the entire image (e.g. horizontal fibre bundles in the centre of Figure 3A).

Chemical fixation shows a homogenous preservation throughout the entire specimen. The quality matches the expectation of this technique with a decent tripartite rendering of the lipid bilayer (LB in Figure 3) and all classic elements that characterise a desmosome. The inserting keratin filaments and the dense plaque (DP in Figure 3) are clearly discernable and the transition zone between both reveals some structural details that slightly differ between unstretched (Figure 3A) and stretched cells (Figure 3B). The inner parts of the dense plaque (toward the nucleus) appear in stretched cells slightly less dense and spine‐like protrusions occasionally show (e.g. lower panel of Figure 3B). However, the expected stretch‐induced unfolding of desmoplakin cannot be identified. Cadherins spanning the intercellular gap are occasionally visible in the unstretched cell but are also discernable in the stretched cells (red arrows in Figure 3). The desmosome of an 80% stretched high‐pressure frozen cell shows a clearly better structural preservation. The lipid bilayer can undoubtedly be traced for most of the membrane profile and cadherins spanning the intercellular gap between the cells are better recognisable as compared to conventional fixation. Most impressively, the zone of overlapping cadherins in the intercellular gap is clearly visible (cyan arrow).

### 3.2 Comparison of virtual sections from EM tomography

Figure 4A shows a desmosome after HPF of unstretched cells grown on a sapphire disc. In the main panels of Figure 4, two virtual sections (thickness: 3.8 nm, distance between both sections along the *z*‐axis: 30 nm) of the reconstructed tomogram are depicted. The series of thresholded images below show the indicated areas of the above main panels (1 and 8) and all consecutive sections between them (2–7). The desmosomal cadherins that span the intercellular space between the two cells are exquisitely well preserved. The cadherins (probably aggregates of several) appear as clearly discernable stalactite/stalagmite‐like structures that can be traced throughout several sections in the desmosomal gap, as indicated by the green and red arrows (see threshold images in Figure 4A, sections 3–8). In the desmosome the outermost parts of the extracellular cadherin domains from opposing cells overlap in the intercellular space, providing the physical linkage between both cells. By HPF on sapphire disks even these overlapping parts withstood the preparation procedure and appear as a thin (partly interrupted) electron dense band in the middle of the intercellular gap (see left main panel in Figure 4A and cyan arrows in threshold images sections 1–3). Also the lipid bilayer in sapphire‐HPF cells shows excellent structural preservation. The characteristic two electron dense lines encasing the brighter lipophilic core of the lipid bilayer can clearly be distinguished and stretches for a considerable length along the membrane profile. The inner part (toward the nucleus) of the dense plaque that contains the potential mechanosensor desmoplakin showed a slightly jagged transition zone. Since desmoplakin is the linker between the keratin filaments and the other dense plaque proteins, the inward pointing spikes within this transition zone might represent (parts of) the desmoplakin.

Figure 4B shows a desmosome grown on the PDMS membrane after HPF with same panel arrangement as in Figure 4A. Compared to the sapphire‐HPF cells we observed rather drastic differences in the ultrastructural preservation (freezing artefacts) within the same sample or even sample region. Figure 4B demonstrates that these freezing artefacts can occur at a distance of a few hundred nanometres from apparently less affected regions. The upper cell shows the typical honeycomb pattern of freezing artefacts whereas the lower cell is obviously much less affected. Except some small areas with suspiciously thick (clotted) electron dense material (sections 1, 2, 3, 4, lower third of insert), ultrastructural preservation is close to HPF on sapphire discs in these regions. Cadherin that bridges the desmosomal gap can be recognised and tracked over several sections (red and green arrows in sections 1, 2, 3, 4), although slightly less crisp. Also, the good preservation of the lipid bilayer demonstrates the relatively good quality of structural preservation in some regions. The dense plaque looks more homogenous as compared to HPF on the sapphire disc and the transition zone between dense plaque and keratin filaments appears slightly less detailed. However, also here the spike‐like protrusions are still discernable, although slightly thicker possibly caused by clotting of its molecular components.

## 4 DISCUSSION

Techniques in electron microscopy have steadily improved in the last decades with HPF‐immobilisation probably being one of the approaches with the best structural preservation and minimal fixation artefacts. This process not only requires elaborate sample preparation, but also the growth support for the preceding steps in cell culture needs to fit into the holder of the HPF apparatus. Hence cells are usually cultured on a rigid sapphire disc (diameter: 6 mm) that fits the holder and provides optimal thermal transfer rates during the HPF process. For conventional cell culture only minor modification of the cell culture procedures are needed to grow cells on these discs. However, in many experimental instances a rigid and air‐/watertight growth support is not suitable, for example, when organotypic culture conditions are simulated. Also experiments where the properties of the growth support need to be altered (e.g. cell stretch) practically exclude the use of sapphire discs to cultivate the cells. Different alternatives to sapphire discs have been introduced (summarised in Jiménez et al.^13^); however, none of them would be suitable for cell stretch experiments.

In our experiments we investigate the mechano‐response of cells that have been cultivated on elastic silicone membranes. The membrane is stretched to stimulate the cells and often needs to remain stretched for further analysis including EM sample preparation that involves dehydration and resin embedding. Since superb ultrastructure is required to elucidate the ultrastructural change upon stretch of the desmosomal protein desmoplakin, we decided to perform HPF and developed a clamping device that allows to mount the stretched sample in the holder of the HPF apparatus. Our study was not intended to prove stretch‐induced ultrastructural changes of desmoplakin but to evaluate a newly developed method that can be used in future experiments.

Briefly the membrane is clamped between a base plate and a ring (Figure 2) and protected with a lid prior to mounting it into the holding bracket that is then inserted in the HPF apparatus to perform HPF (see also Figure 1).

During the development process, the following technical necessities and challenges were encountered and had to be addressed.

The tight fixation of the membrane depends predominantly on the backlog of the ring/base plate (Figure 2C, orange oval). On the one hand, a tight fit is required to fasten the membrane, on the other hand the edges of the ring/base plate should not sever the membrane when the ring is pressed into the base plate. Latter requires a gap between the overhanging edges of ring/base plate of not less than approximately 50 µm. Due to inconsistencies in the manufacturing process the workshop of the university produced three different dimensions with slightly increasing gap widths and the best (50 µm) was chosen. To minimise the risk of severing the membrane, the part of the ring applicator that holds the base plate (grey cylinder under the membrane in Figure 1F–H) was not tightly screwed but had a lateral give of approximately half a millimetre to compensate for non‐centric lowering of the plunger.

Another problem was encountered when removing the membrane clip (Figure 1I) from the holding bracket after HPF. Frozen leftovers of the silicone membrane held the membrane clip tightly in the holding bracket. Hence we had to increase the inner diameter of the holding bracket by 300 µm for a looser fit. Alternatively, also the outer diameter of the membrane clip could have been reduced.

During the HPF process, the cells need to be protected with a lid that easily detaches later when the sapphire disc is used. For unknown reasons the lid did not detach from the membrane clip and we had to gently remove it manually with a scalpel (still submersed in liquid nitrogen) after HPF.

When comparing the ultrastructure of HPF‐immobilised cells with cells after conventional chemical fixation (both grown on the silicone membrane) an improvement in structural details could be observed (Figure 3). Especially the clearly discernable overlapping extracellular cadherin domains (cyan arrows in Figure 3C) indicated very good structural preservation in the HPF‐immobilised cells, when compared to chemical fixation. Here the overlap of cadherin domains is barely visible and the extracellular domains appear truncated (Figure 3A and B, red arrows). No overlapping part in the intercellular gap was observed. Also, the two dark hydrophilic parts of the lipid bilayer are better preserved and appear more distinct in the HPF‐immobilised specimen.

However, we also noticed that the quality of the HPF process varies locally and larger areas with obvious freezing artefacts (large honeycomb patters) can be observed in close vicinity to areas with better preservation within the same sample. This heterogenicity is well captured in Figure 4, where virtual sections of HPF‐immobilised cells grown on sapphire discs (Figure 4A) are compared with cells grown on unstretched membranes (Figure 4B). The upper cell in Figure 4B (section 1 and overview on the right panel) clearly shows the above‐mentioned honeycomb pattern, with a surprising improvement of image preservation in the lower cell. There are smaller regions with freezing artefacts also in the lower cell though (three honeycombs in the lower third area of the insert). Interestingly these freezing artefacts become less apparent when looking at section 8 (distance along the *Z*‐axis: ca. 30 nm). Apparently, the heterogeneous preservation not only occurs within the *XY*‐plane but also along the *Z*‐axis of the sample. This is in line with reports from a group using Aclar discs as growth support for HPF.^13^ Aclar is a copolymer film with similar characteristics as cell culture plastic. Even when using cryoprotectants, not all cells on the Aclar disc were properly frozen. We can only speculate about the reasons that cause the inhomogeneous freezing process in our samples, but the combination of the elastic silicone membrane with the custom made HPF clip is certainly the most important aspect. The substantially different design as compared to conventional HPF with the sapphire disc might have affected proper vitrification by altered heat transfer rates of the entire unit. The relatively deep indentation of the lid (800 µm, see Figure 2A) and the hole in the base plate (HO in Figure 2A) might partly account for it. However, we believe that the different heat transfer by the PDMS membrane is the most likely candidate to explain the freezing artefacts. Taken together, even excluding the parts of the specimen with apparent freezing artefacts the superb image quality of the cell on the sapphire disc was not entirely achieved with our method. However, membranes after HPF‐immobilisation show better structural preservation as compared to chemical fixation.

Our study demonstrates, that even stretched elastic membranes can be used for HPF‐immobilisation. Ultrastructural preservation exceeds chemical fixation although HPF‐immobilised cells on sapphire discs clearly outranks both. The inhomogeneous freezing of the HPF‐immobilised cells on the membrane still needs to be addressed and alternative designs for the membrane clip might might be necessary to further improve ultrastructural preservation. Since various cell culture techniques depend on non‐solid growth supports that are easier to handle than stretched membranes (e.g. permeable foils for air–liquid–interface cultures) the authors are confident that the new technique can be applied for various cell culture techniques other the cell stretch.
